# Soluble silica stimulates osteogenic differentiation and gap junction communication in human dental follicle cells

**DOI:** 10.1038/s41598-020-66939-1

**Published:** 2020-06-18

**Authors:** Pamela Uribe, Anders Johansson, Ravin Jugdaohsingh, Jonathan J. Powell, Catarina Magnusson, Marcela Davila, Anna Westerlund, Maria Ransjö

**Affiliations:** 1 Department of Orthodontics, Institute of Odontology, The Sahlgrenska Academy, University of Gothenburg, Gothenburg, Sweden; 2 Unit of Molecular Periodontology, Department of Odontology, University of Umeå, Umeå, Sweden; 3 Biomineral Research Group, Department of Veterinary Medicine, University of Cambridge, Cambridge, UK; 4 The Bioinformatics Core Facility at the University of Gothenburg, Gothenburg, Sweden

**Keywords:** Preclinical research, Biomaterials - cells

## Abstract

Several studies have indicated that dietary silicon (Si) is beneficial for bone homeostasis and skeletal health. Furthermore, Si-containing bioactive glass biomaterials have positive effects on bone regeneration when used for repair of bone defects. Si has been demonstrated to stimulate osteoblast differentiation and bone mineralisation *in vitro*. However, the mechanisms underlying these effects of Si are not well understood. The aim of the present study was to investigate the effects of soluble Si on osteogenic differentiation and connexin 43 (CX43) gap junction communication in cultured pluripotent cells from human dental follicles (hDFC). Neutral Red uptake assay demonstrated that 25 μg/ml of Si significantly stimulated hDFC cell proliferation. Dosages of Si above 100 μg/ml decreased cell proliferation. Alizarin Red staining showed that osteogenic induction medium (OIM) by itself and in combination with Si (25 μg/ml) significantly increased mineralisation in hDFC cultures, although Si alone had no such effect. The expression of osteoblast-related markers in hDFC was analysed with RT-qPCR. *OSX, RUNX2, BMP2, ALP, OCN, BSP* and *CX43* genes were expressed in hDFC cultured for 1, 7, 14 and 21 days. Expression levels of *BMP-2* and *BSP* were significantly upregulated by OIM and Si (25 μg/ml) and were also induced by Si alone. Notably, the expression levels of *OCN* and *CX43* on Day 21 were significantly increased only in the Si group. Flow cytometric measurements revealed that Si (50 μg/ml) significantly increased CX43 protein expression and gap junction communication in hDFC. Next-generation sequencing (NGS) and bioinformatics processing were used for the identification of differentially regulated genes and pathways. The influence of OIM over the cell differentiation profile was more prominent than the influence of Si alone. However, Si in combination with OIM increased the magnitude of expression (up or down) of the differentially regulated genes. The gene for cartilage oligomeric matrix protein (COMP) was the most significantly upregulated. Genes for the regulator of G protein signalling 4 (RGS4), regulator of G protein signalling 2 (RGS2), and matrix metalloproteinases (MMPs) 1, 8, and 10 were also strongly upregulated. Our findings reveal that soluble Si stimulates Cx43 gap junction communication in hDFC and induces gene expression patterns associated with osteogenic differentiation. Taken together, the results support the conclusion that Si is beneficial for bone health.

## Introduction

Silicon (Si) is a trace element that is suggested to be essential for the normal development of connective tissues and the skeleton^1–3^. Dietary Si is mainly found in plant-based foods, such as rice, whole-grain cereals (including cereal products such as beer), vegetables, fruit, and mineral water. Absorption of Si depends on its chemical form, with the soluble orthosilicic acid (OSA, Si(OH)_4_) being the most bioavailable. The absorption of Si in the intestine is followed by a rapid increase in the serum levels of Si, and Si that is not retained by the body is excreted in the urine within 4–8 h^4^. Early studies of Si depletion in chicks and rats reported severely impaired growth and abnormal development of the skeletal and connective tissues^5,6^. Although more recent studies have failed to repeat these dramatic effects of Si deprivation in animals, there is strong evidence that Si is positively correlated with bone metabolism and homeostasis. Interestingly, young rats that were fed a Si-depleted diet showed increased longitudinal growth due to inhibition of growth plate closure and differences in calcium to phosphorus ratio^7^. In the Framingham offspring cohort, strong positive correlations between dietary Si intake and hip bone mineral density was demonstrated. The effect was most pronounced in pre-menopausal women and less so in men and absent in post-menopausal women. This indicated a relationship between oestrogen status and the effects of Si on bone health, which was confirmed in the Aberdeen Prospective Osteoporosis Screening Study (APOSS)^8,9^. The role of soluble Si in bone formation has been investigated at the cellular level, and Si was demonstrated to stimulate osteoblast differentiation, extracellular matrix synthesis, and increased bone mineralisation *in vitro*^3,10–13^. In line with these findings, it has been demonstrated that biomaterials that contain Si, e.g., bioactive glasses such as 45S5 Bioglass, stimulate osteoblast differentiation, as evidenced by increased expression of the osteoblastic markers osteocalcin and alkaline phosphatase and an increase in new bone formation^14–16^. However, the mechanisms underlying the effect of Si on osteogenic differentiation and bone formation are not well understood.

The dental follicle (DF) is an ectomesenchymal tissue sac that surrounds the un-erupted tooth. It appears to regulate osteoclastogenesis and osteogenesis needed for the eruption process^17–19^. Pluripotent mesenchymal stem cells have earlier been demonstrated to be present in the DFs of human wisdom teeth^20^ and in murine DFs^21^. Previous studies have demonstrated that stimulated DF cells (DFC) can form mineralised nodules *in vitro*, and that DFC express bone-related proteins, such as alkaline phosphatase (ALP), bone sialoprotein (BSP) and osteocalcin (OCN)^20,22–24^. Human DFC (hDFC) are easily accessible and do not entail the same ethical concerns and controversies as embryonic stem cells. The ability of hDFC to differentiate into different cell types makes them good candidates to study osteoinductive materials, as well as an alternative source of stem cells for tissue regeneration^20^.

The aim of the present *in vitro* study was to clarify the effects of soluble Si on osteogenic differentiation and bone formation using hDFC. We investigated the effects of Si on gene expression and bone nodule formation (matrix mineralisation) in hDFC compared to osteogenic induction media (OIM). We used next-generation sequencing (NGS) and bioinformatics processing to determine the transcriptomic profiles of hDFC that were cultured in the absence or presence of OIM and Si, alone or in combination. Furthermore, the effects of Si on Connexin 43 (CX43) expression and gap junction communication (GJC) in hDFC were assessed, since Cx43-mediated GJC is crucial for osteoblast differentiation and bone formation^25–27^.

## Patients and Methods

### Ethics

All experiments and methods were performed in accordance with relevant guidelines and regulations. All experimental protocols were approved by the Regional Ethics Board at the University of Gothenburg (Dnr. 898–13) and by the National Data Inspection Board. Informed consent was obtained from the patients and their parents. The methods described below have been reproduced in part from Uribe *et al*.^28^

### Establishment of cell cultures of hDFC

hDFC cultures were established from dental follicles obtained from four different patients with impacted canines who were referred for orthodontic reasons. The patients ranged in age from 12 to 16 years. After being rinsed in Minimum Essential Medium α (α-MEM) (Gibco, Life Technologies, USA), the dental follicle tissues were minced using a sterilised scalpel, and placed in culture plates with α-MEM that was supplemented with 10% (v/v) foetal bovine serum (FBS), 2 mM Glutamax, and an antibiotic-antimycotic solution (all from Gibco, Life Technologies) at 37 °C in humidified atmosphere consisting of 5% CO_2._ After 48 hours of incubation, non-adherent cells were removed by changing the growth medium. The adherent cells were cultured and maintained with medium change every 3 days. Passages 3–5 were used for all the experiments.

### Preparation of Silica solutions

The Si-containing medium used in the experimental setup was prepared using the method of Sripanyakorn *et al*.^29^, with minor alterations^28^. A stock solution (350 μg/ml) was prepared by adding 0.1 ml of concentrated sodium silicate (Sigma-Aldrich) to 49.9 ml of α-MEM. The pH was adjusted to 7.0–7.2 using HCl before 10% FBS (v/v) (Gibco Invitrogen) was added. Solutions with final concentrations of 0–250 μg/ml Si were prepared by diluting the stock solution with α-MEM + 10% FBS.

### Cell proliferation assay

Neutral Red (NR) uptake assays were used to estimate cell proliferation, as previously described by Repetto *et al*.^30^. hDFC were cultured for 48 h in 96-well plates with 100 μl growth medium (α-MEM and 10% FBS) per well and in the absence or presence of 0–250 μg/ml Si. NR dye (Merck, Dannstadt, Germany) was dissolved in PBS (4 mg/ml), diluted 1:100 in α-MEM, and incubated for 24 h at 37 °C to obtain the NR incubation medium (4 μg/ml). The NR incubation medium was centrifuged at 200 × *g* for 5 min prior to usage and added 100 μl/well. After 2 h of incubation at 37 °C, the cells were washed with PBS (150 μl/well), and the NR destaining solution (150 μl/well; 10 ml H_2_O, 10 ml EtOH 99.5%, and 0.2 ml glacial acetic acid) was added to release NR from the lysosomes in the cells. After 10 min, the absorbance of the solubilised dye was quantified at 540 nm in a spectrophotometer multi-plate reader (Multiskan FC Microplate Photometer; Fisher Scientific). Protocol validated previously by Uribe *et al*.^28^. The experiment was repeated three times with four replicates per treatment.

### Osteogenic induction medium

To determine osteogenic differentiation capabilities, hDFC were grown in 24-well plates to confluency. Thereafter, the hDFC were cultured for 1, 7, 14 and 21 days with osteogenic induction medium (OIM) that contained α-MEM, 10% FBS, 50 mg/ml L-ascorbic acid 2-phosphate sesquimagnesium salt and 10 mM β-glycerophosphate disodium salt hydrate, with or without Si (25 μg/ml). Untreated cells cultured in α-MEM were used as controls.

### Alizarin red staining

Alizarin Red S (ARS) staining of hDFC cultures was used to study the effects of Si on bone mineralisation. ARS is a dye that binds selectively to calcium salts and is widely used for calcium mineral histochemistry. As previously described by Saugspier *et al*.^31^, monolayers of hDFC cultured for 1, 7, 14, 21 and 28 days with OIM ± Si were washed twice with PBS, fixed with ice-cold 70% ethanol for 30 min, and stained for 30 min with 40 mM ARS (Sigma Aldrich, St. Louis, MO, USA), at pH 4.2 at room temperature with rotation on an orbital shaker. The samples were then rinsed five times with water to reduce non-specific ARS staining and allowed to air-dry. Stained cells were photographed and this was followed by a quantitative extraction method adapted from the protocol described by Stanford *et al*.^32^. Briefly, ARS was released from the cell matrix by incubation of the monolayers with 10% (w/v) cetylpyridinium chloride (CPC) in 10 mM sodium phosphate (pH 7.0) for 1 h (1 ml/well). The ARS extracts were then removed and 100-μl aliquots were transferred in to a 96-well plate prior to reading the absorbance at 562 nm in a multi-plate reader (Multiskan FC Microplate Photometer; Fisher Scientific). Control values were obtained from control samples without cells. The experiment was repeated three times with four replicates per treatment.

### Real-time quantitative polymerase chain reaction (RT-qPCR)

The Minimum Information for Publication of Quantitative Real-Time PCR Experiments (MIQE) guidelines were followed to ensure the relevant experimental conditions and assay characteristics^33^. RNA extraction was carried out using the RNeasy Plus Mini Kit (Qiagen, Hilden, Germany) according to the manufacturer’s instructions. Briefly, cell monolayers were rinsed with PBS and lysis buffer was added to each well. RNA was bound to the silica membrane using ethanol. The column was rinsed and washed with the TR2 and TR3 buffers, and DNase I treatment was performed to remove any contaminating DNA. The RNA was eluted, and then stored at −20 °C. The RNA concentrations were quantified with the Qubit Fluorometer (Invitrogen, Burlington, ON, Canada).

All the reverse-transcription steps were performed using the iScript cDNA Synthesis Kit (Bio-Rad Laboratories, Hercules, CA, USA) with 1 μg of total RNA. Two microliters of Universal RNA Spike (TATAA Biocenter, Gothenburg, Sweden) were added to each sample to allow quality control throughout the entire RT-qPCR experimental workflow as previously described in Uribe *et al*.^28^.

To select the most stable reference genes for normalization, a panel of 12 reference genes was screened in 5 samples from every group and time-point. The expression profiles of the screened reference genes were evaluated using the geNorm^34^ and Normfinder^35^ programs. The *HPRT1* and *GUSB* genes showed the most stable expression among the samples and were therefore selected as the reference genes for the subsequent analyses. The measured Cq value and the shape of the amplification curve revealed no inhibition in the presence of RNA spiking in the control assays.

The primers used in the RT-qPCR were purchased from Bio-Rad Laboratories (Table 1). The analysis of the target genes and the two selected reference genes was performed in a 10-μl reaction volume (10 ng of cDNA per reaction) in duplicate on a CFX 96 Real-Time System (Bio-Rad Laboratories) using the SsoAdvanced Universal SYBR Green Supermix (Bio-Rad Laboratories). An inter-plate calibrator (TATAA Biocenter) was added to each plate to compensate for the variation between runs. The quantities of the target genes were normalised using the geometric mean of the Cq values of the selected reference genes. Gene expression was quantified according to the comparative threshold cycle method Δ − ΔCq and 90% PCR efficiency^36^.

**Table 1:** Bio-Rad SYBR Green primers used for the RT-qPCR analyses.

Gene identification	Abbreviation	Unique Assay ID
**Target gene encoding:**		
Sp7 transcription factor	OSX	qHsaCED0003759
Runt-related transcription factor 2	RUNX2	qHsaCED0044067
Bone morphogenetic protein 2	BMP2	qHsaCID0015400
Alkaline phosphatase	ALP	qHsaCID0010031
Gap junction protein, alpha 1, 43 kDa	CX43	qHsaCID0012977
Bone gamma-carboxyglutamate protein (BGLAP)	OCN	qHsaCED0038437
Integrin-binding sialoprotein	BSP	qHsaCED0002933
Reference gene encoding:		
β-Glucuronidase	GUSB	qHsaCID0011706
Hypoxanthine phosphoribosyltransferase 1	HPRT1	qHsaCID0016375

### CX 43 protein expression and Functional analysis of gap junctions

The methods described below have been reproduced in part from Uribe *et al*.^28^ To quantify the expression of the CX43 protein, hDFC were prepared as a cell suspension and stained for analysis with flow cytometry. Briefly, hDFC were cultured with or without Si (50 µg/ml) for 48 h. Cells were detached with ice-cold EDTA with the aid of a cell scraper. Aliquots of 10^6^ cells in 100 µl of α-MEM were stained and labelled with a human connexin43/GJA APC-conjugated antibody (monoclonal mouse IgG_2A_ Clone 578618, R&D systems) for 20 min at 4 °C. Thereafter, the cells were washed twice with PBS, and finally suspended in 500 µl of PBS. An isotype b antibody (Mouse IgG2A Allophycocyanin conjugated, R&D systems) with a different spectrum of detection than the antibody of interest was used as a control.

Intercellular communication via gap junctions was quantified using the fluorescent dye transfer parachute technique described by Czyz *et al*.^37^. Briefly, hDFC were cultured for 3 days in 12-well plates in α-MEM medium that contained 10% FBS, in the absence or presence of 25 μg/ml Si. For the donor cells, a cell suspension from a parallel culture was prepared with a staining solution using two dyes: the membrane permeable green dye calcein-AM (1 μg; Molecular Probes Inc., Eugene OR, USA); and the lipophilic red dye Dil (1 mM; Molecular Probes), used at final concentrations of 2 μM and 10 mM, respectively, for 30 min at 37 °C. The stained cells were washed with PBS and centrifuged three times (200 × *g* for 5 min), and thereafter re-suspended in 1 ml PBS with 2% FBS. Then, 2% of the double-stained donor cells were added to the unstained recipient cells at a ratio of 1:50 (donor:recipient) and incubated at 37 °C in 5% CO_2_ for 1, 2 and 3 h. Carbenoxolone (CBX) was added as an inhibitor of GJC, and used as a negative control. A parallel plate was placed on ice before the donor cells were added, to allow blocking of GJC, and used as a negative control. The non-fluorescent dye calcein-AM is hydrolysed by intracellular esterases into the fluorescent calcein, and can, thereafter, only pass from the donor to recipient cells through functional gap junction channels. Second-, third-and higher-order cells will acquire the dye depending on the degree of coupling. GJC is quantified by flow cytometry. The flow cytometer detected Dil-stained cells by FL3 and calcein-AM-stained cells by FL1. Donor cells were detected as cells with high intensities for both FL1 and FL3, while the recipient cells were distinguished by their increased intensity of FL1. Flow cytometry analysis has a high sensitivity and provides an appropriate resolution that allows the detection of small changes in gap-junctional communication by examining a large number of cells in a single experiment. The gating strategy for the parachute analyses is based on location of donor cells and recipient cells incubated on ice. The gated area should include as few donor cells as possible, but include the recipient cells with increased color uptake. This strategy is based on the original study by Czyz *et al*.^35^. By gating the populations, the recipient cells could be identified and quantified.

### Next-generation sequencing

Whole-transcriptome RNA sequencing using next-generation sequencing (NGS-RNAseq) technologies and bioinformatics was used to compare the gene expression levels in hDFC cultured in the absence or presence of OIM and Si. RNA was collected from hDFC that had been cultured for 1, 7, 14 and 21 days with OIM with or without Si (25 μg/ml). Untreated cells cultured in α-MEM were used as controls. RNA extraction was carried out with the RNeasy Plus Mini Kit (Qiagen) according to the manufacturer’s instructions, and the samples were frozen at −80 °C before sending for library preparation.

The quality control (Trimmomatic), alignment (TopHat), and differential expression analysis (Cuffdiff) were performed at GATC (https://www.gatc-biotech.com). The resulting data were further analysed using R (ver. 3.4.2,^38^). Graphical visualisations, such as heat maps and functional profiles, were created using the pheatmap (ver. 1.0.8) and goProfiles (ver. 1.40.0).

### Statistical analysis

The gene expression data were analysed using PRISM 7 (GraphPad Software Inc., San Diego, CA, USA). Analysis of variance (ANOVA) was performed to test for significant differences in gene expression and absorbance percentages between controls and treated groups. Statistical significance was adjudged for *p*-values ≤0.05. Each experiment was repeated three times with four biological replicates per treatments, unless otherwise stated in legends to figures.

## Results

### Cell viability and proliferation

**Figure 1:**
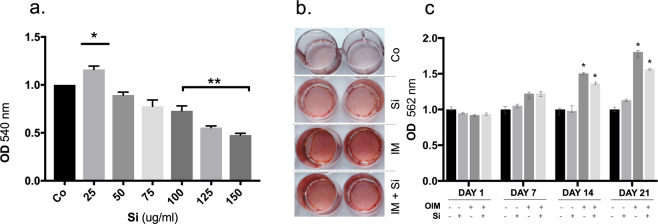
Effects of Si and OIM on proliferation and mineralization in cultures of hDFC. (**a**) Neutral Red uptake by hDFC cultured for 48 h with different concentrations of Si. Data are normalised to control, where the control (Co) absorbance values are set to 1. Values presented are mean ± SEM (n = 3). (**b**) Representative images of nodule mineralisation in hDFC cultured for 21 days in the absence or presence of Si (25 µg/ml) and OIM, and then stained with Alizarin Red. (**c**) Quantification of calcium deposition after 21 days. OIM has a significant effect on the mineralisation process after 21 days of culture. A significant effect of calcium deposition is observed in the presence of Si (25 µg/ml) plus OIM after 14 days and 21 days, as compared to the control cells. Data are normalised to control, where the control absorbance values are set to 1. Values shown are the averages of three different experiments (n = 15). *p ≤ 0.001.

hDFC were cultured with different concentrations of Si for 48 h, Cell viability and proliferation was anlysed using the neutral red (NR) assay. A dose-dependent change in NR uptake was demonstrated (Fig. 1a). 25 μg/ml Si caused a significant (*p* = 0.001) stimulatory effect on cell proliferation. No significant effects on of Si was seen at concentrations >25 and up to 75 μg/ml, as compared to the control cells. A significant decrease in the number of viable hDFC was seen when cells were cultured with Si ≥100 μg/ml (*p* < 0.0001) (Fig. 1a)

### Mineralised nodule formation

Alizarin Red staining (ARS) was used to determine the formation of mineralised nodules in hDFC cultures exposed to osteogenic induction medium (OIM) and Si. OIM stimulated the formation of mineralisation nodule in cultured hDFC, as revealed by ARS staining. The effect of Si on ARS staining was small and Si did not have any additive effect when combined with OIM (Fig. 1b). When ARS release from the matrix was determined, a significant stimulatory effect of OIM (*p* = 0.0102) on the mineralisation process was seen after 14 and 21 days. There was no additive effect of Si (25 μg/ml) in combination with OIM (*p* = 0.0063). There was no significant effect of Si alone on ARS/nodule formation at any time-point (Fig. 1c).

### Gene expression analyses

**Figure 2:**
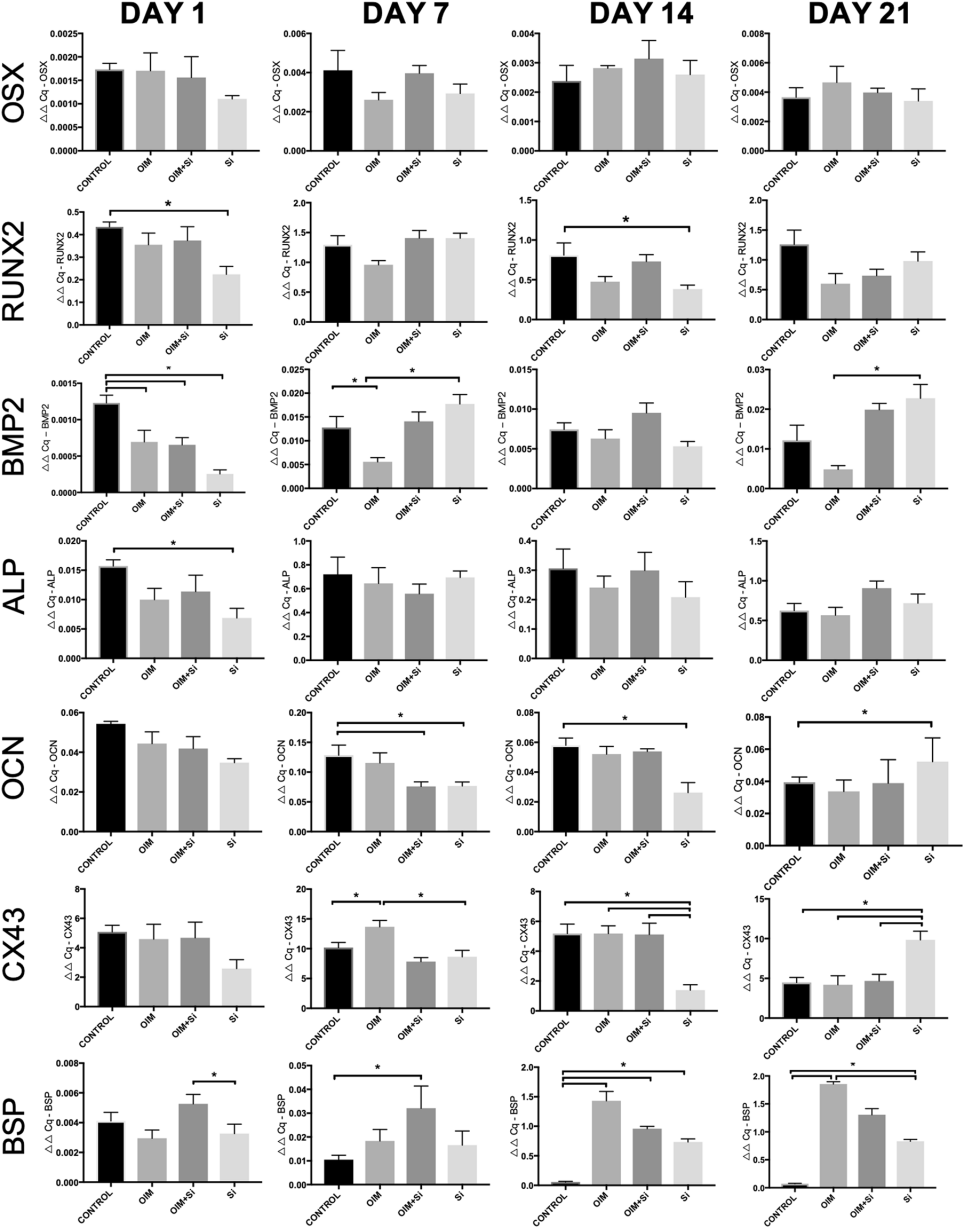
Effects of Si on mRNA levels of osteoblastic phenotypic markers expressed in hDFC. Expression levels of markers of osteoblastic differentiation in hDFC that were cultured for 1, 7, 14, and 21 days in the absence or presence of Si (25 µg/ml) and OIM. Presented are the relative values normalised to *HPRT1* and to the control. Values shown are mean ± SEM (n = 3). *p ≤ 0.01.

The expression levels of the osteoblast markers *OSX*, *RUNX2*, *BMP2*, *ALP*, *OCN*, and *BSP* and the gap junction gene *CX43* was investigated with Quantitative RT-qPCR in hDFC cultured for 1, 7, 14 21 days in the presence or absence of OIM and Si (25 µg/ml) (Fig. 2). All the above-listed genes were expressed in control cultures of hDFC and also in the cells cultured in the presence of OIM and/or Si (25 µg/ml) for 21 days. The expression levels were low on Day 1, except for *CX43* which was highly expressed in all groups at Day 1, however, no statistically significant differences were seen between groups. Significantly (*p* = 0.001) increased expression of *BMP2* and *OCN* were demonstrated in hDFC exposed to Si or to Si plus OIM for 21 days, as compared to controls. A time-dependent significant increase in *BSP* levels (*p* < 0.0001) was seen in the OIM treated groups. *CX43* was significant up-regulated after 21 days, but only in the Si-treated group (*p* = 0.0054), (Fig. 2). The expression of *BMP2* was significantly higher in the Si group, as compared to the OIM group on Day 7 (*p* < 0.0001) and Day 21 (*p* = 0.0025). *BSP* was significantly higher expressed in the OIM + Si group than in the other groups on Day 7 (*p* = 0.001). *BSP* was significantly higher in the OIM + Si (*p* < 0.0001) and Si (*p* < 0.0001) groups on Day 14,, as compared to the control group. The expression level was highest with OIM alone (*p* < 0.0001 and this was also seen on Day 21. (Fig. 2).

### Next-generation sequencing (NGS-RNAseq)

**Figure 3:**
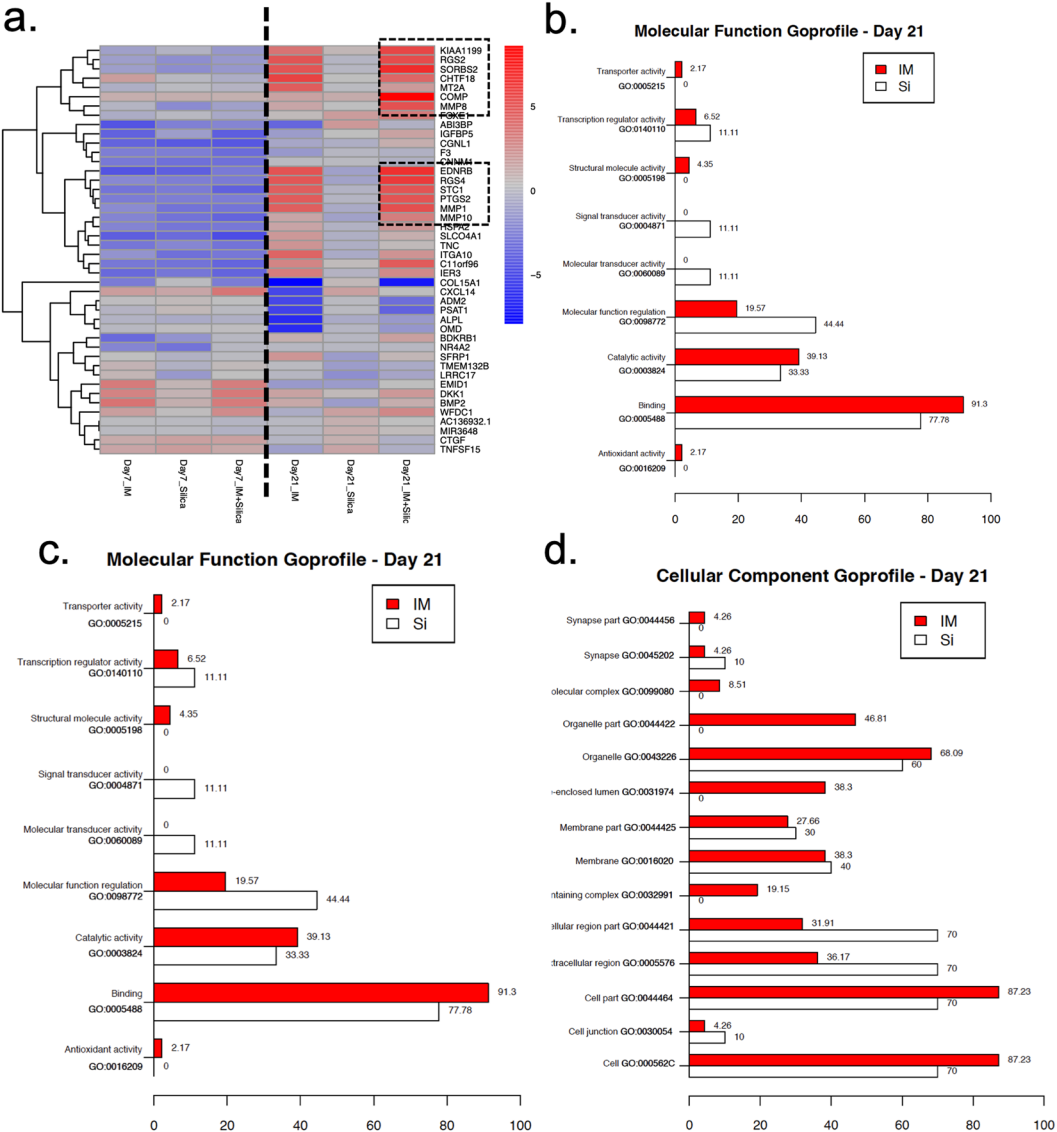
Impacts of Si on gene expression in hDFC during osteoblastic differentiation. (**a**) Heat map representation of medium-dependent effects on hDFC that were cultured for 7 and 21 days in growth medium that contained OIM, Si, or OIM + Si. Genes enclosed by a discontinuous line are highly expressed and regulated by the presence of Si during osteoblastic differentiation. (**b, c, d**) The gene ontology of hDFC after 21 days of differentiation regulated by OIM and Si.

The heat map of expression data shown in Fig. 3a demonstrates the impacts of OIM, OIM + Si and Si alone on gene expression in hDFC. Regardless of the group examined, two main gene expression patterns were noted: (i) a pattern representative of cells cultured for 7 days (Fig. 3a, left); and (ii) a pattern representative of cells cultured for 21 days (Fig. 3a, right). hDFC that were cultured with OIM and OIM + Si clustered together, while those cultured with Si alone formed a separate branch. These results demonstrate the influence that OIM exerts on the cell differentiation profile, which is stronger than the influence of Si alone. However, Si in combination with OIM showed an augmented effect on the differentially regulated genes that were subjected to hierarchical cluster analysis, which is in line with the results previously reported for the functional test by matrix deposition and PCR quantification of osteoblast-related markers. The ten most highly up-regulated genes, as well as the three most down-regulated genes are listed in Table 2. Cartilage oligomeric matrix protein (COMP) was the most significantly up-regulated gene. Genes for the regulator of G protein signalling 4 (RGS4), regulator of G protein signalling 2 (RGS2), and matrix metalloproteinases (MMP) 1, 8, and 10 were also highly up-regulated (Table 2). Regulated genes were further clustered according to gene functions and according to their associated biological processes, annotated to gene ontology (GO) terms. The GO profiles for hDFC in the presence of Si or in OIM medium are listed in terms of molecular functions (Fig. 3b), biological processes (Fig. 3c), and cellular components (Fig. 3d). Most of the clustered genes were regulated by both Si and OIM. However, the molecular function gene-sets for signal transducer activity and molecular transducer activity were significantly differentially expressed only in hDFC cultured with Si alone.

**Table 2:** The most highly up- and down-regulated genes in hDFC induced to undergo osteoblastic differentiation.

Ensembl ID	Gene symbol	Gene name	Fold-change	p-value
***Up-regulated genes***				
ENSG00000105664	COMP	cartilage oligomeric matrix protein	8.58	0.013
ENSG00000154556	SORBS2	sorbin and SH3 domain-containing 2	6.60	0.013
ENSG00000136160	EDNRB	endothelin receptor type B	6.43	0.013
ENSG00000117152	RGS4	regulator of G protein signalling 4	5.99	0.024
ENSG00000103888	KIAA1199	Cell migration inducing hyaluronidase 1	5.50	0.013
ENSG00000116741	RGS2	regulator of G protein signaling 2	5.23	0.013
ENSG00000196611	MMP1	matrix metalloproteinase 1	5.14	0.013
ENSG00000118113	MMP8	matrix metalloproteinase 8	5.02	0.031
ENSG00000159167	STC1	stanniocalcin-1	4.93	0.013
ENSG00000073756	PTGS2	prostaglandin-endoperoxide synthase 2	4.93	0.013
ENSG00000178919	FOXE1	forkhead box E1	3.26	0.038
ENSG00000166670	MMP10	matrix metalloproteinase10	3.04	0.013
ENSG00000103175	WFDC1	WAP four-disulfide core domain 1	2.68	0.038
ENSG00000137331	IER3	immediate early response 3	2.62	0.013
***Down-regulated genes***				
ENSG00000128165	ADM2	adrenomedullin 2	−3.08	0.013
ENSG00000135069	PSAT1	phosphoserine aminotransferase 1	−3.66	0.013
ENSG00000204291	COL15A1	collagen type V alpha 1 chain	−6.89	0.013

### Cx43 expression and Gap Junction communication

**Figure 4:**
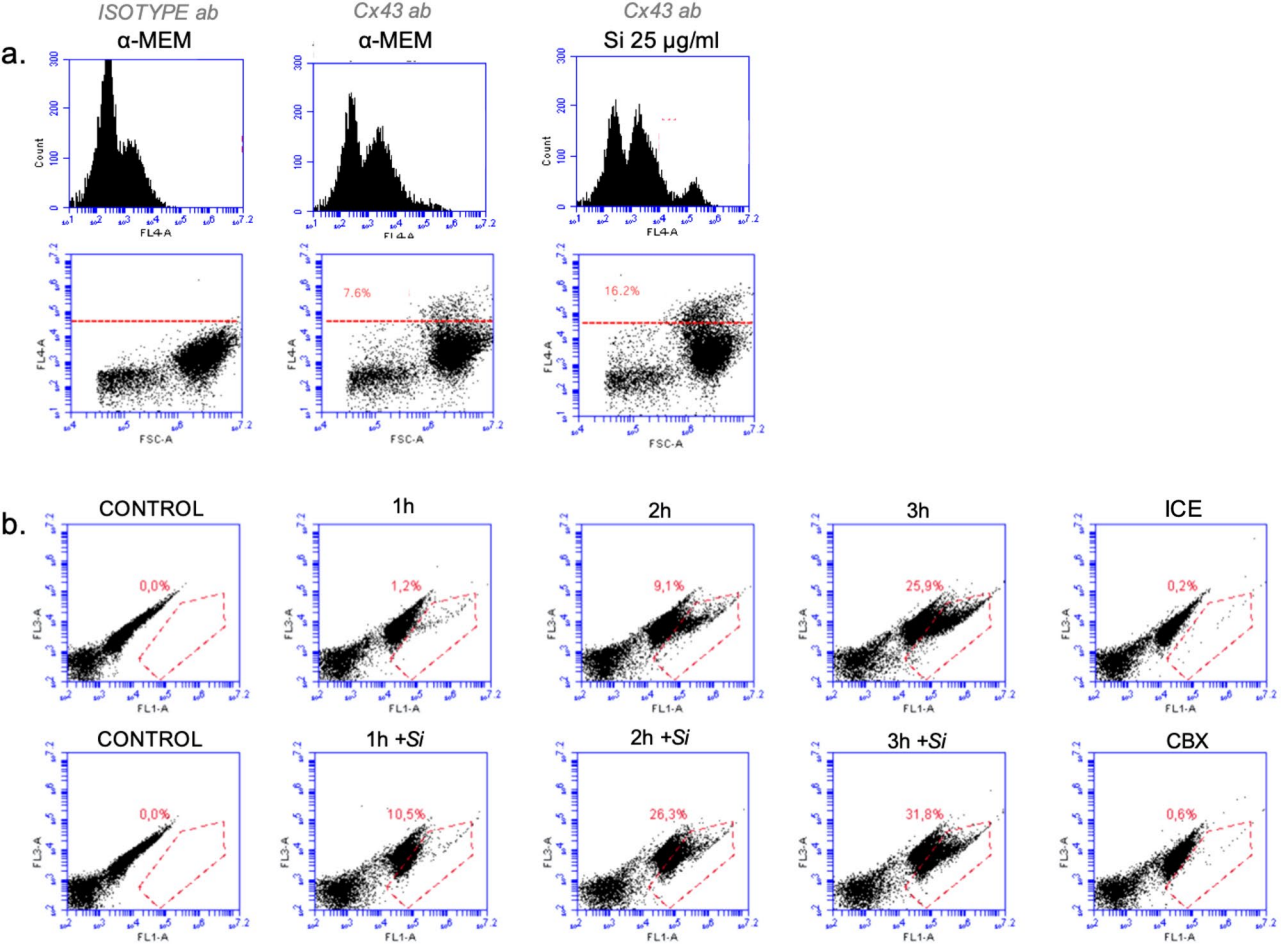
CX43 protein expression and functional analysis of gap junction communication in hDFC. (**a**) Expression of CX43 protein in hDFC cultured for 48 h with or without Si (25 µg/ml) presented as dot plots and histograms. Representative graphs and proportion of recipient cells are shown. (**b**) Flow cytometry of the coupling ratio in hDFC. Gating strategy for the percentage of recipient-positive cells after 1 h, 2 h and 3 h with or without Si (25 µg/ml). Donor cells are characterised by high intensities of green (FL1) and red (FL2) fluorescence; recipient cells are identified by colouration with the green transferred dye (FL1). Representative graphs and proportion of recipient cells are shown.

Flow cytometry quantification of the surface expression of CX43 protein revealed that a small fraction of hDFC express CX43 (7.6%) on their surface. An increase in the expression of CX43 (16.2%) was observed after exposure to Si (25 μg/ml) for 48 h (Fig. 4a). GJC in the hDFC was then analysed using the parachute dye-loading technique. Control cells were incubated at 37 °C in α-MEM and the results demonstrate an increased dye-uptake in the recipient cells, which indicate an active GJC communication (Fig. 4b). The presence of Si (25 μg/ml) in the culture medium progressively increased GJC after 1, 2, and 3 h of incubation, resulting in a significant increase in the numbers of stained-recipient hDFC compared to the corresponding controls without Si treatment (Fig. 4b). This outcome strongly suggests that Si increases the GJC function. The transfer of dye between the donor and recipient cells was limited when the cells were kept on ice, and dye transfer was totally blocked by the gap junction blocking compound CBX, demonstrating that the dye transfer is due to GJC activity.

## Discussion

Earlier studies have demonstrated that dietary Si is important for wound healing as well as for bone homeostasis^1–3^ confirming its important role in connective tissues. Si has been reported to be taken up from the plasma and incorporated into bovine and rat bones, although the amount of Si in human bone tissue is not yet established^39,40^. The mechanisms of action of soluble Si on bone remain unclear, although it has been demonstrated that Si enhances prolyl hydroxylase activity, stabilizes collagen cross-linking, and increases bone matrix mineralisation *in vitro*^1,3,8,9,41^. To date, it has not been established if the effects of Si are mainly extracellular. However, direct regulatory effects of Si on osteoblast activities have been demonstrated. Furthermore, we have recently reported that soluble Si inhibits osteoclast formation and bone resorption *in vitro* by interacting with both inter- and intra-cellular pathways^42^. Si transporters have been demonstrated in rice and in diatoms. The Lsi1 transporter in rice is a membrane protein with similarities to mammalian aquaporins^43^. Even if there is still no evidence that Si can be transported into bone cells, there are some indications that Si is transported into mammalian cells, as provided by the finding that Slc34a2, a sodium-phosphate co-transporter, acts as an active Si transport protein in rat kidney cells^44^ that appears to be involved in the reabsorption of Si from the pre-urine.

Si-containing biomaterials, e.g., bioactive glasses, promote bone regeneration when used to treat local bone defects^14–16,45^. The effects have been attributed to locally released ionic dissolution products (Si, Ca and P;^46,47^), although it is possible that physical and chemical properties and ions other than Si are also involved^48^. The concentrations of Si released from bioactive glass 45S5 into cell culture medium, that we previously determined with inductively coupled plasma optical emission spectrometry (ICP-OES), were used in the present study.^42^. We used hDFC from dental follicles of impacted teeth as the source of mesenchymal pluripotent cells, to investigate the process of osteogenic induction with soluble Si. Multipotent adult stem cells from dental tissues represent a potential source of mesenchymal stem cells for experimental studies, as well as for clinical treatments to repair local bone defects. It has previously been demonstrated that hDFC have a higher proliferation ability than mesenchymal stem cells derived from the pulp^49^. hDFC forms an alternative pool of cells that can easily be obtained from impacted teeth, since the follicle is usually otherwise discarded following surgical procedures.

A small proliferation was demonstrated when the hDFC were cultured in the presence of Si at 25 µg/ml. This concentration also had stimulatory effects on the differentiation markers in long-term experiments and therefore used in most of the subsequent experiments. The results also showed that Si at concentrations ≥100 μg/ml had inhibitory effects on cell proliferation. One reason for this is that Si at high concentrations (above 2 mM or 50–60 μg/ml) autocondenses to form small polymers^50^ that are less bioavailable but also interacts readily with cell membranes. Thus, the inhibitory effects of higher concentrations of Si seen in the present study could be due to the presence of polymeric forms of Si, which would likely have negative effects on cell growth.

OIM has earlier been demonstrated to promote osteoblast phenotypic differentiation, as evidenced by the increased expression of late osteoblastic markers, collagen accumulation, and ALP activity in osteoblast-like cells^51^. In line with these findings, exposure to an osteogenic differentiation environment, such as that with ascorbic acid and β-glycerol phosphate or growth factors (BMP-2 and TGFβ1), has previously been demonstrated to induce osteogenic differentiation of DFC^24,31^. Our present results confirm that OIM itself has osteogenic effects, as demonstrated by significant stimulation of matrix mineralisation and increased expression of the *BSP* gene. Although the increase in matrix mineralisation could not be demonstrated in hDFC cultured with Si alone, there was significant up-regulation of the expression of *BMP2* and *BSP* in hDFC treated with only Si. In contrast, the expression of *BMP2* was not increased with OIM alone, but in combination with Si. Our results suggests that although Si does not have the capacity by itself to support matrix mineralisation, it can stimulate osteogenic differentiation in hDFC. This role is supported by BMP2 which plays an important role in the regulation of osteogenic differentiation^52^. In line with this, increased expression of *OCN* was only demonstrated in the Si group. NGS-RNAseq analysis and bioinformatics processing were used to identify differentially regulated genes and pathways. The results demonstrate that effect of OIM over the differentiation profile was more significant than the effects of Si alone. However, Si in combination with OIM had an additive effect (increase) on the expression of the differentially regulated genes. Interestingly, *COMP* was the most significantly up-regulated gene. COMP expression had earlier been demonstrated in MG-63 cells and is suggested to be important for osteogenesis^53^. Our results are in line with those earlier reports of a significant up-regulation of both *BMP2* and *COMP* during osteogenic differentiation of human dental follicle stem cells^54^. Our results further demonstrate that the genes for regulator of G protein signalling 4 (RGS4), regulator of G protein signalling 2 (RGS2), and matrix metalloproteinases (MMPs) 1, 8, and 10 are also highly up-regulated in hDFC undergoing osteogenic differentiation. The RGS proteins are important for the regulation of G protein-coupled receptors and they influence important signalling pathways in many tissues, including bone. RGS2 has been reported as being expressed in osteoblasts and induced by the cAMP-PKA pathway. The transcriptome analysis in our present study thus supports the notion that Si could have a regulatory role in the signalling pathways involved in osteogenic differentiation and bone formation.

GJC is an important signaling system which contributes to the differentiation of cells and tissues by transferring ions and second messengers between adjacent cells. It has earlier been demonstrated that CX43 and gap junction channels in bone are crucial for osteoblast differentiation, bone matrix formation, and the normal mineralization of the bone tissue^55^. Moreover, osteoblast dysfunction, craniofacial defects, and a delayed skeletal mineralization, is reported in CX43-null mice^26,56^. In addition, we, and others, have earlier demonstrated the importance of CX43 and GJC for osteoclast formation and for the bone resorbing activities of osteoclasts^55,57,58^. We have recently reported *CX43* gene expression in human dental follicles^59,60^. A novel finding in the present paper is our results demonstrating significantly higher *CX43* gene and protein expression and GJC in Si-treated hDFC. These findings suggest that signals that are dependent upon GJC are crucial during the differentiation process regulated by Si. We used a dye-transfer assay to evaluate the possible functional role of Si on GJC in hDFC. The GJC activity in hDFC was significantly stimulated by Si. This suggest that GJC could be a signaling pathway to mediate effects of Si in osteogenic differentiation. The results demonstrating that OIM has no effect on *CX43* gene expression agree with Hashida *et al*.^27^. They reported an important role for CX43 GJC in BMP2-induced osteoblast differentiation, and furthermore, that CX43 GJC did not mediate the osteogenic effects by ascorbic acid. This is interesting since our results in the present study validate that Si, but not OIM, increase the expression of BMP2.

Even though Si by itself is not sufficient to initiate a mineralisation process, our results show that Si can stimulate factors and signals important for osteoblastic differentiation. This strongly suggests that dietary Si is beneficial for bone formation and skeletal health. Furthermore, it has been reported that Si-containing biomaterials stimulate bone regeneration when used for repair of bone defects. Gaining evidence of the mechanisms involved with Si interactions on osteogenic differentiation is necessary to better use Si-containing biomaterials in the clinical practice. Furthermore, our results point to an interesting opportunity to use undifferentiated mesenchymal cells obtained from human dental follicles for the validation of osteoinductive materials and for bone regeneration.

## Supplementary information

Supplementary Information.

Supplementary Information2.
