# Supplementary material for: Soluble silica stimulates osteogenic differentiation and gap junction communication in human dental follicle cells
Source: Sci Rep. 2020 Jun 18;10:9923. doi: 10.1038/s41598-020-66939-1 (PMC7303172; doi:10.1038/s41598-020-66939-1)
Supplement: Supplementary file 1 — Supplementary Information. [file 41598_2020_66939_MOESM1_ESM.docx]

# soluble silica stimulates osteogenic differentiation and gap junction communication in human dental follicle cells

**Pamela Uribe^a^, Anders Johansson^b^, Ravin Jugdaohsingh^c^, Jonathan J. Powell^c^, Catarina Magnusson^a^, Marcela Davila^d^, Anna Westerlund^a^ and Maria Ransjö^a^**

^a^ Department of Orthodontics, Institute of Odontology, The Sahlgrenska Academy, University of Gothenburg, Gothenburg, Sweden

^b^ Unit of Molecular Periodontology, Department of Odontology, University of Umeå, Sweden.

^c^ Biomineral Research Group, Department of Veterinary Medicine, University of Cambridge, Madingley Road, Cambridge, CB3 0ES, UK.

^d^ The Bioinformatics Core Facility at the University of Gothenburg, Gothenburg, Sweden

**Correspondence to:**

Pamela Uribe, Department of Orthodontics, Institute of Odontology, The Sahlgrenska Academy, University of Gothenburg, PO Box 450, 405 30 Gothenburg, Sweden. E-mail: [pamela.uribe@gu.se](mailto:pamela.uribe@gu.se) , puribe@ces.edu.co
