# Supplementary material for: Soluble silica stimulates osteogenic differentiation and gap junction communication in human dental follicle cells
Source: Sci Rep. 2020 Jun 18;10:9923. doi: 10.1038/s41598-020-66939-1 (PMC7303172; doi:10.1038/s41598-020-66939-1)
Supplement: Supplementary file 2 — Supplementary Information2. [file 41598_2020_66939_MOESM2_ESM.docx]

**Key resources table.**

| **Reagent** | **Source** | **Identifier** |
| --- | --- | --- |
| **Chemicals, peptides and recombinant proteins** | | |
| Minimum Essential Medium α | (Gibco, Life Technologies, USA |  |
| Foetal bovine serum (FBS) | Gibco, Life Technologies |  |
| Glutamax | Gibco, Life Technologies |  |
| Antibiotic-antimycotic solution | Gibco, Life Technologies |  |
| Concentrated sodium silicate | Sigma Aldrich, St. Louis, MO, USA |  |
| Neutral Red Dye | Merck, Dannstadt, Germany |  |
| L-ascorbic acid 2-phosphate sesquimagnesium salt | Sigma Aldrich, St. Louis, MO, USA |  |
| β-glycerophosphate disodiumsalt hydrate | Sigma Aldrich, St. Louis, MO, USA |  |
| Alizarin Red Staining | Sigma Aldrich, St. Louis, MO, USA |  |
| cetylpyridinium chloride (CPC) | Sigma Aldrich, St. Louis, MO, USA |  |
| Sodium phosphate | Sigma Aldrich, St. Louis, MO, USA |  |
| RNeasy Plus Mini Kit | Qiagen, Hilden, Germany |  |
| iScript™ cDNA Synthesis Kit | Bio-Rad Laboratories, Hercules, CA, USA |  |
| Universal RNA Spike | TATAA Biocenter, Gothenburg, Sweden |  |
| SsoAdvanced™ Universal SYBR® Green Supermix | Bio-Rad Laboratories, Hercules, CA, USA |  |
| APC-conjugated anti-GJA1 antibody | Inc., San Diego, CA, USA |  |
| Calcein-AM | Molecular Probes Inc., Eugene OR, USA |  |
| dye Dil | Molecular Probes |  |
| Carbenoxolone | Sigma Aldrich, St. Louis, MO, USA |  |
| **Primers** | | |
| OSX, Sp7 transcription factor | Bio-Rad Laboratories, Hercules, CA, USA | qHsaCED0003759 |
| RUNX2, Runt-related transcription factor 2 | Bio-Rad Laboratories, Hercules, CA, USA | qHsaCED0044067 |
| BMP2, Bone morphogenetic protein 2 | Bio-Rad Laboratories, Hercules, CA, USA | qHsaCID0015400 |
| ALP, Alkaline phosphatase | Bio-Rad Laboratories, Hercules, CA, USA | qHsaCID0010031 |
| CX43, Gap junction protein, alpha1, 43 kDa | Bio-Rad Laboratories, Hercules, CA, USA | qHsaCID0012977 |
| OCN, Bone gamma-carboxyglutamate protein | Bio-Rad Laboratories, Hercules, CA, USA | qHsaCED0038437 |
| BSP, Integrin-binding sialoprotein | Bio-Rad Laboratories, Hercules, CA, USA | qHsaCED0002933 |
| GUSB, β-Glucuronidase | Bio-Rad Laboratories, Hercules, CA, USA | qHsaCID0011706 |
| HPRT1, Hypoxanthine phosphoribosyltransferase 1 | Bio-Rad Laboratories, Hercules, CA, USA | qHsaCID0016375 |
